# Reversible leukoencephalopathy with seizures: a case of severe high-altitude cerebral edema

**DOI:** 10.1186/s42494-024-00165-4

**Published:** 2024-07-18

**Authors:** Ting Chen, Xintong Wu, Xiaoyan Liu, Fengming Luo

**Affiliations:** 1 Department of High Altitude Medicine, Center for High Altitude Medicine, West China Hospital, Sichuan University, 37 Guo Xue Xiang, Chengdu, Sichuan 610041 China; 2 Department of Neurology, West China Hospital, Sichuan University, 37 Guo Xue Xiang, Chengdu, Sichuan 610041 China; 3 Department of Neurology, Lhasa People’s Hospital, 1 Beijing Middle Road, Lhasa, Xizang, 850000 China

**Keywords:** High-altitude cerebral edema, Reversible leukoencephalopathy, Microbleeds, Seizures, Case report

## Abstract

**Background:**

Acute high-altitude illness (AHAI) refers to a series of syndromes including acute mountain sickness (AMS), high-altitude pulmonary edema (HAPE) and high-altitude cerebral edema (HACE). Among these, HACE is a severe and potentially life-threatening condition that can occur when individuals ascend to high altitudes. It is often characterized by ataxia, confusion, and altered mental status. Without appropriate treatment, HACE can rapidly progress to coma, but seizures are infrequent in occurrence.

**Case presentation:**

Here, we report a severe HACE patient with coma and status epilepticus. The patient is a 23-year-old male who was visiting Lhasa for the first time. He initially experienced headaches and dizziness on the first day, and then he was found in coma with limb convulsions on the next day. Immediate medical attention was sought, and brain CT and MRI scans showed reversible white matter lesions, especially in the corpus callosum and subcortical white matter. Although the lesions disappeared on T1 and T2 sequences, microbleeds were observed on the SWI sequence. After treatment with tracheal intubation, glucocorticoids and hyperbaric oxygen, the cerebral edema has resolved and the clinical symptoms improved, the patient has no seizures anymore.

**Conclusions:**

HACE typically follows AMS and poses a significant risk to life. Clinical manifestations mainly include ataxia, alterations of behavior, and impaired consciousness, with severe cases progressing to coma. Seizures, though rarely observed, may occur. Imaging shows reversible white matter lesions, with microbleeds being a significant and persistent imaging marker over time. Administration of glucocorticoids plays a crucial role in treatment. Despite experiencing seizures, this patient did not experienced any further episodes once his condition improved.

## Background

Acute high-altitude illness (AHAI) encompasses a range of syndromes, including acute mountain sickness (AMS), high-altitude pulmonary edema (HAPE), and high-altitude cerebral edema (HACE). These conditions occur when individuals are unable to adapt to the environment shortly after ascending to high altitudes (above 2500 m), where they are exposed to low barometric pressure and reduced oxygen levels (hypoxia) [1]. HACE is a severe and potentially life-threatening condition that is often considered as a late or end-stage of AMS. If not promptly diagnosed and managed, HACE can rapidly progress to coma and death as a result of brain herniation within 24 h [2]. HACE is often characterized by ataxia, confusion, and changes in mental status but seizures are rare [1, 3]. Here, we report a severe HACE patient with coma and status epilepticus.

## Case presentation

**Fig. 1:**
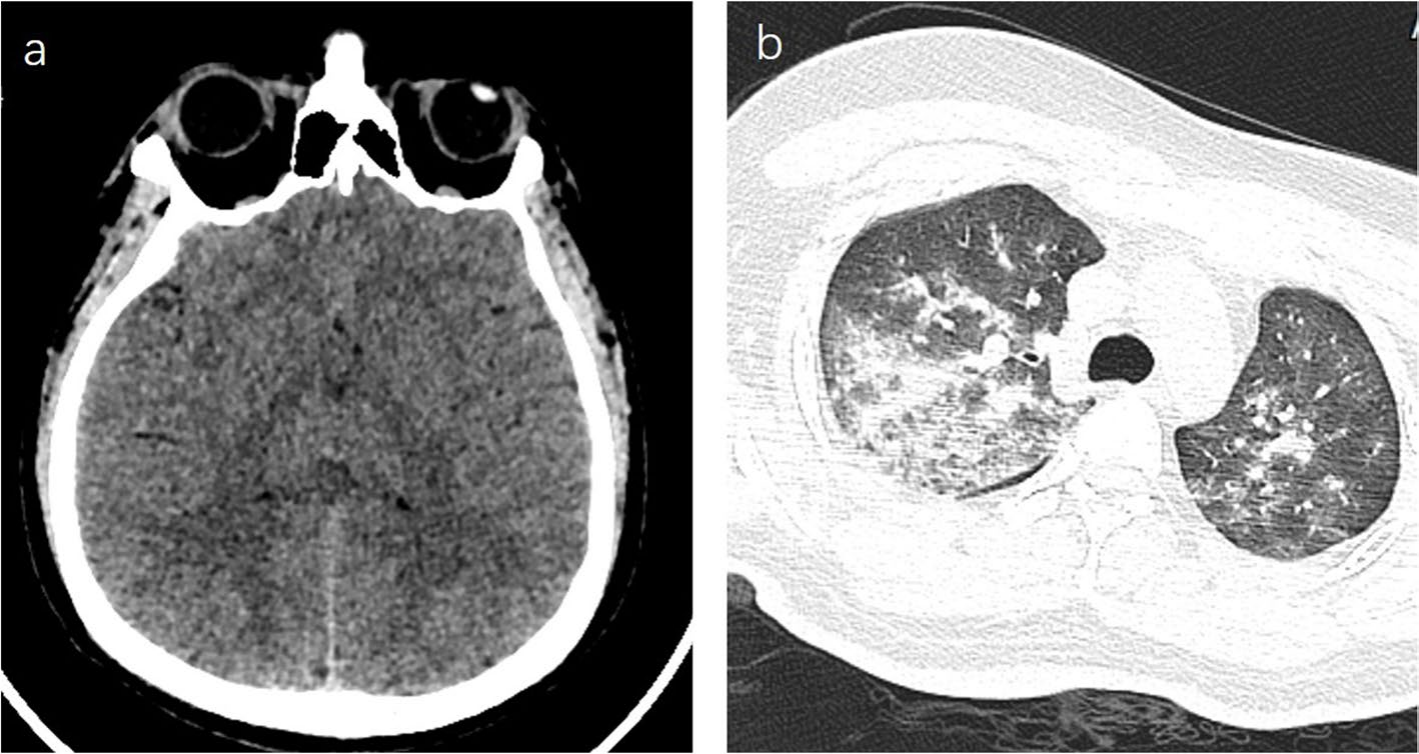
The CT scan conducted at the local hospital in Lhasa revealed cerebral edema and pulmonary edema. **a** The cortical sulci and gyri have disappeared, and the boundary between gray and white matter is indistinct. **b** Bilateral patchy edema in both lungs, particularly prominent on the right side

A 23-year-old male from Jiangxi province, China, who was sent to the emergency department of West China hospital on May 26, 2023, with a chief complaint of “headache, dizziness for 5 days, worsening with coma and convulsion for 4 days”. The patient had traveled to Lhasa for the first time by train on May 21, 2023, and soon after arrival, he experienced headache and dizziness. Following the administration of oxygen, he went to his accommodation for rest. However, on May 22, 2023, he was found unconscious with generalized convulsions, prompting immediate transfer to a local hospital in Lhasa. Computed tomography (CT) scans of his head and chest revealed cerebral edema (Fig. 1a) and pulmonary edema (Fig. 1b). The patient presented with status epilepticus, leading to interventions such as tracheal intubation, intracranial decompression, sedation and administration of dexamethasone. He was then urgently transferred to the emergency department of our hospital, where he remained comatose and was subsequently admitted to the neuro-intensive care unit (NICU) on May 30, 2023. The treatment regimen involved mannitol for dehydration to alleviate intracranial pressure, dexamethasone to mitigate brain edema, along with anti-infection and anti-seizure measures. The tracheal intubation was successfully removed on June 2, and the patient was transferred to our high-altitude medical department on June 8.

### Physical examination

The patient’s vital signs were stable, general medical examination and cranial nerve assessments revealed no abnormalities. However, there were abnormalities observed in his long-term memory and calculations (eg., 100 -7 = 92). Muscle tone, strength, and most coordination tests were within normal limits. Nonetheless, the patient had difficulty completing the heel-knee-shin test, and Romberg’s sign was positive. Pathological reflexes, including Babinski’s sign and meningeal irritation sign, were negative.

### Auxiliary examinations

**Fig. 2:**
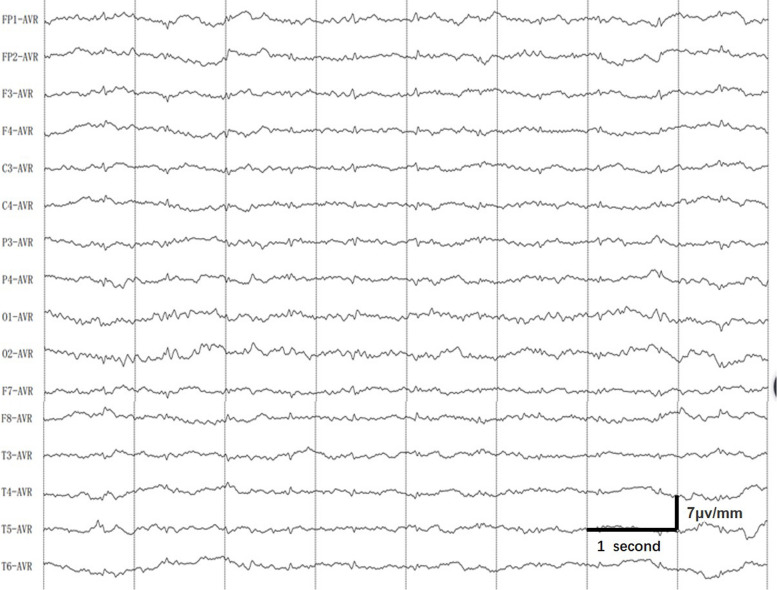
EEG shows a background of bilateral fast waves with an increased presence of slow waves

**Fig. 3:**
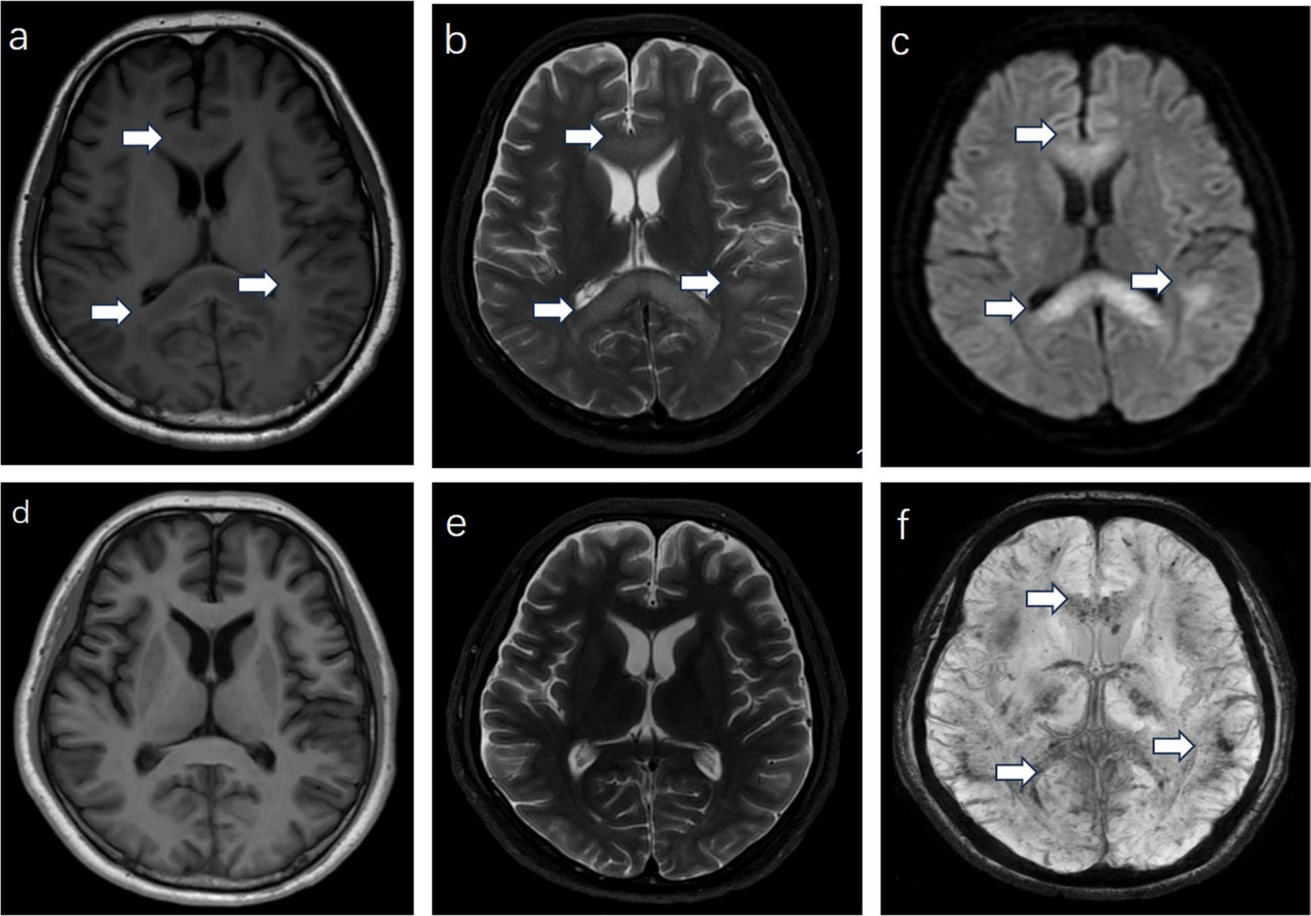
The follow-up brain MRI. There were T1W1 low signals (**a**, white arrow), T2W1 high signals (**b**, white arrow), and DWI high signals (**c**, white arrow) in the corpus callosum, knee region, and junction of the cortex and medulla on June 1st. On June 20th, compared to the June 1st scan, the abnormal signals observed in the T1W1 and T2W1 sequences had significantly decreased (**d**, **e**). SWI sequence showed diffuse micro-bleedings (**f**, white arrow)

A lumbar puncture was performed, revealing an opening pressure of 195 mmH_2_O. The cerebrospinal fluid (CSF) showed an increased protein concentration of 0.58 g/L (normal range: 0.15–0.45g/L). However, other CSF parameters, including virus, bacteria and autoimmune encephalitis antibodies etc, were within normal limits. The patient's performance on the mini-mental state examination (MMSE) and Montreal cognitive assessment (MoCA) scales, revealed scores of 17 and 16 points, respectively, on June 19. The electroencephalogram (EEG) showed diffuse slow activity in the background with bilateral fast activity (Fig. 2). On May 26, the re-examination of the head and chest CT scans exhibited signs of cerebral parenchymal swelling, reduced density of the white matter, and subtle bilateral ventricular constriction. Pulmonary assessments revealed scattered light patchy and striped shadows in both lungs, coupled with mild consolidation in the lower lobes. Subsequent brain magnetic resonance imaging (MRI) on June 1 revealed pronounced abnormal T1 and T2 signal in the corpus callosum and the bilateral white matter (Fig. 3). On June 9, the re-examination of the brain MRI revealed a notable reduction in the size of the lesions. A chest CT scan re-evaluation delineated scattered inflammation in both lungs, with diminished absorption in comparison to previous examinations. On June 20, the brain MRI indicated complete disappearance of the lesions on T1 and T2 sequences. However, the susceptibility-weighted imaging (SWI) unveiled dot-like paramagnetic signals in the corpus callosum and the bilateral gray-white matter junction, suggesting diffuse intracranial micro-hemorrhage (Fig. 3).

Upon admission to the high-altitude medical department, the patient complained persistent memory decline, mild headaches and an occasional cough, with no seizures, dizziness, nausea, vomiting, or shortness of breath. Following symptomatic supportive treatment including hyperbaric oxygen therapy, the patient’s symptoms improved significantly and he was discharged on June 21.

## Discussion

Altitude regions are usually defined as intermediate altitude (1500–2500 m), high altitude (2500–3500 m), very high altitude (3500–5800 m), extreme altitude (> 5800 m) and the “Death zone”(> 8000 m) [4]. With the development of the economy and transportation, an increasing number of people are traveling, working, or engaging in competitive sports at high altitudes. Therefore, altitude sickness has emerged as a public health concern with corresponding economic impacts [5]. AMS is the most frequently observed high-altitude illness, often benign and self-limiting. In contrast, HACE and HAPE occur rather rarely but pose life-threatening risks [6, 7].

The primary risk factors for the developing high-altitude illness include the rate of ascent, the altitude reached (especially the sleeping altitude), the degree of pre-acclimatization, and individual susceptibility. Other risk factors for high-altitude illness include a history of high-altitude illness and permanent residence lower than 900 m [2]. Contrary to popular belief, physical fitness does not offer protection against high-altitude disease [8]. It has been estimated that 25% of people at moderate altitude are affected by AMS, and 50–85% of travelers at 4000 m or or higher are affected [9, 10]. The incidence of HACE and HAPE is much lower than that of AMS. At altitudes ranging from 4000–5000 m, the incidence rate of HACE is about 0.5–1%, affecting individuals of different genders and ages. although younger males may be at a higher risk due to continued ascent despite symptoms of AMS and a faster rate of ascent [3]. HACE often occurs 2 days after reaching an altitude of exceeding 4000 m, but it can also occur earlier or in the areas above 2500 m [6]. Before developing into HACE, some patients may experience AMS symptoms such as headache, insomnia, anorexia, nausea, and some may also be accompanied by HAPE. However, HACE can also arise without preceding symptoms of AMS and has also been reported to present acutely within a matter of hours. Research has found that 50% of patients who die from HAPE also suffer from HACE. Therefore, among patients diagnosed with HACE, HAPE is a common occurrence [11]. If not promptly recognized and treated, HACE may rapidly evolve into coma with fatal consequences.

Our patient, a young man with no prior exposure to high-altitude environments, initially developed symptoms of AMS, characterized by headache and dizziness, which rapidly progressed to HAPE and HACE [12]. However, it is worth noting that he experienced seizures and even status epilepticus, which are uncommon in cases of HACE. There has been a report of new-onset seizures occurring at high altitude without signs of AMS or HACE [13]. Additionally, a study has demonstrated when individuals residing at sea level and subsequently exposed to high altitudes face an elevated risk of experiencing new-onset seizures in the immediate few months following exposure, with this risk increasing in correlation with higher altitudes [14]. However, there are limited reports of seizures occurring in HACE. Unfortunately, EEG was not conducted when the patient experienced status epilepticus at the first hospital. Subsequent EEG performed in our department during the absence of seizure activity revealed diffuse slow activity, indicating cortical functional impairment. So lumbar puncture was performed in order to rule out other potential diagnoses such as infections, autoimmune encephalitis and other diseases [15, 16].

The pathogenesis of cerebral edema involves the abnormal accumulation of water in the brain parenchyma, affecting both the interstitium and intracellular compartments [17]. Different types of altered (or impaired) brain water regulation, each with a distinct physiological process, have been identified. There are three major types of brain edema: intracellular, ionic, and vasogenic [12]. Brain imaging studies have revealed signs of intracellular edema within cerebral white matter following acute hypoxic exposure, particularly in individuals with AMS. Although evidence supporting the existence of brain edema is not common, long periods of hypoxemia (> 22 h) have provided the most convincing evidence of increased white matter volume in persons with AMS, suggesting a pathological link between AMS and HACE [18]. Intracellular edema may occur early, while subtle extracellular edema (ionic or vasogenic) only begins to develop after 16 to 22 h, which coincides with the peak of AMS symptomology in controlled chamber experiments and marks the timeframe when HACE can potentially begin to develop [19]. In the early stages of hypoxemia, diffusion-weighted imaging (DWI) may show increasing signal intensity, and the apparent diffusion coefficient (ADC) images may exhibit reduced signal intensity, suggesting the presence of intracellular edema. With prolonged periods of hypoxemia, ionic edema is further detectable as hyperintensity on T2WI or fluid-attenuated inversion recovery (FLAIR) images, usually affecting both white and grey matter, and accompanied with restricted diffusion on DWI images and reduced signal on ADC [17, 20]. Vasogenic edema, on the other hand, can be identified by hyperintensity on T2 and FLAIR images without restriction on DWI. Neuroimaging of this patient presented widespread cerebral edema, with particular involvement of the corpus callosum and white matter, representing its primary features [21, 22]. As the cerebral edema situation and the symptoms significantly improved, these lesions appeared reversible on T1 and T2 sequences of brain MRI, suggesting as reversible leukoencephalopathy. However, the presence micro-bleeding lesions on SWI did not dissipate. A prevailing theory is that hypoxia results in severe vasodilation leading to elevated capillary pressure and leakage [23]. The HACE patients shows obvious hemosiderin depositions in the brain, especially in corpus callosum. There are relevant case reports indicating that even after 10 years from the onset, micro-bleeding lesions can still be observed, serving as evidence of prior HACE occurrence [21, 22]. These microbleeds only exist in individuals diagnosed with HACE and have not been observed in cases of AMS or isolated HAPE [24].

Due to the significant morbidity and mortality associated with HACE, prevention should be prioritized over treatment [25, 26]. It is likely that both AMS and HACE share a common pathological process, similar measures should be utilized for the prevention of both conditions. The non-pharmacologic preventive strategies includes slow ascent and pre-acclimatization. When at moderate altitude (2200–3000 m), individuals should avoid increasing their sleeping elevation by > 300–600 m per night and should incorporate a rest day every 3–4 days, during which they spend an additional night at the same elevation [5]. This remains the best method for HAI prevention. The pharmacologic prevention such as acetazolamide may be an effective preventive measure and can also be used to treat mild AMS, however, it is not recommended for treatment of moderate to severe AMS or HACE. The recommended dosage for adults is 250 mg every 12 h and for children, it is 2.5 mg per kg (maximum: 250 mg) every 12 h [6, 27].

Regarding the treatment of HACE, several measures should be implemented, including administering oxygen therapy with increased inspired oxygen fraction, bringing the patient to a lower altitude, using a hyperbaric chamber to immediately decreases cerebral blood flow and intracranial pressure, and employing drugs to prevent the formation of cerebral edema. But the use of the corticosteroids is considered as a crucial and reliable treatment for moderate to severe AMS or HACE. The recommended dexamethasone regimen for adults with HACE involves an initial dose of 8 mg administered orally, intravenously, or intramuscularly, followed by 4 mg every 6 h until symptoms resolve [1, 5, 27]. Our patient was treated with corticosteroids at both the local hospital in Lhasa and our hospital, along with hyperbaric oxygen treatment, which played a vital role in his recovery.

## Conclusions

We report a case of a typical HACE patient who initially presented with headache and dizziness as AMS. The condition rapidly progressed to coma and was accompanied by acute repetitive seizures with status epilepticus. Clinically, the patient also exhibited signs of HAPE, along with predominantly reversible white matter lesions affecting the corpus callosum, as evident in SWI sequence of brain MRI showing diffuse microbleeds. The patient demonstrated improvement with prompt and effective treatment. Therefore, SWI may serve as an imaging marker for HACE. It is worth noting that seizures in HACE patients tend to subside as symptom improve and typically do not recur.

## Data Availability

The data presented in this study are available on request from the corresponding author.

## Abbreviations

AHAI: Acute high-altitude illness
AMS: Acute mountain sickness
HAPE: High-altitude pulmonary edema
HACE: High-altitude cerebral edema
CT: Computed tomography
MRI: Magnetic resonance imaging
NICU: Neuro-intensive Care Unit
EEG: Electroencephalogram
DWI: Diffusion weighted image
ADC: Apparent diffusion coefficient image
FLAIR: Fluid-attenuated inversion recovery images
CSF: Cerebrospinal fluid
MMSE: Mini-mental state examination
MoCA: Montreal cognitive assessment
AQP4: Aquaporins 4
